# Evaluation of Modulatory Activities of Lactobacillus crispatus Strains in the Context of the Vaginal Microbiota

**DOI:** 10.1128/spectrum.02733-21

**Published:** 2022-03-10

**Authors:** Chiara Argentini, Federico Fontana, Giulia Alessandri, Gabriele Andrea Lugli, Leonardo Mancabelli, Maria Cristina Ossiprandi, Douwe van Sinderen, Marco Ventura, Christian Milani, Francesca Turroni

**Affiliations:** a Laboratory of Probiogenomics, Department of Chemistry, Life Sciences, and Environmental Sustainability, University of Parma, Parma, Italy; b Microbiome Research Hub, University of Parma, Parma, Italy; c APC Microbiome Institute and School of Microbiology, Bioscience Institute, National University of Ireland, Cork, Ireland; d Department of Veterinary Medical Science, University of Parma, Parma, Italy; Università Roma Tre

**Keywords:** *Lactobacillus crispatus*, vaginal microbiota, antibacterial activities

## Abstract

It has been widely reported that members of the genus *Lactobacillus* dominate the vaginal microbiota, which is represented by the most prevalent species Lactobacillus crispatus, Lactobacillus jensenii, Lactobacillus gasseri, and Lactobacillus iners. L. crispatus is furthermore considered an important microbial biomarker due to its professed beneficial implications on vaginal health. In order to identify molecular mechanisms responsible for health-promoting activities that are believed to be elicited by L. crispatus, we performed *in silico* investigations of the intraspecies biodiversity of vaginal microbiomes followed by *in vitro* experiments involving various L. crispatus strains along with other vaginal *Lactobacillus* species mentioned above. Specifically, we assessed their antibacterial activities against a variety of pathogenic microorganisms that are associated with vaginal infections. Moreover, coculture experiments of L. crispatus strains showing the most antibacterial activity against different pathogens revealed distinct ecological fitness and competitive properties with regard to other microbial colonizers. Interestingly, we observed that even phylogenetically closely related L. crispatus strains possess unique features in terms of their antimicrobial activities and associated competitive abilities, which suggests that they exert marked competition and evolutionary pressure within their specific environmental niche.

**IMPORTANCE** The human vaginal microbiota includes all microorganisms that colonize the vaginal tract. In this context, a vaginal microbiota dominated by *Lactobacillus* and specifically by Lactobacillus crispatus is considered a hallmark of health. The role of L. crispatus in maintaining host health is linked to its modulatory activity toward other members of the vaginal ecosystem and toward the host. In this study, *in vitro* experiments followed by genetic analyses of the mechanisms used by L. crispatus in colonizing the vaginal ecological niche, particularly in the production of different antimicrobial compounds, were evaluated, highlighting some intriguing novel aspects concerning the genetic variability of this species. Our results indicate that this species has adapted to its niche and may still undergo adaptation to enhance its competitiveness for niche colonization.

## INTRODUCTION

The human microbiota is the entire set of microorganisms that colonize the host (1–3). Among the latter, scientific interest in the vaginal microbiota (VM) has increased significantly in recent years due to its impact on female health. Several studies have highlighted the substantial diversity of the VM during the human life span, being heavily influenced by hormonal fluctuations (4–7). Immediately following birth, microorganisms originating from the mother and the environment commence colonization of the daughter’s vaginal tract and, at the initial phase of development, the VM is characterized by low levels of lactobacilli and a high level of microbial diversity (8, 9). Subsequently, bacteria belonging to the genus *Lactobacillus* tend to dominate the VM, representing up to 95% of the VM community in healthy women (10, 11). However, both the prevalence and abundance of lactobacilli as well as the distribution of *Lactobacillus* species in the vaginal microbiota are subject to a considerable interindividual variability (11–13). Specifically, five distinct groups of vaginal microbial communities named vaginotypes or vaginal community state types (VCSTs) have recently been proposed (11, 14), with vaginotypes I, II, III, and V characterized by high abundance of Lactobacillus crispatus (LCB), Lactobacillus gasseri, Lactobacillus iners, and Lactobacillus jensenii, respectively (11). It has been proposed that vaginotypes I, II, and III are the most dominant in healthy women (11). Furthermore, VCST IV is characterized by a paucity of lactobacilli, which appear to be replaced by opportunistic bacteria often associated with bacterial vaginosis (15, 16). It seems that not all lactobacilli exert the same health-promoting activities, and recent studies have highlighted that vaginotype I is more strongly associated with vaginal health than the other vaginotypes, thereby suggesting that L. crispatus can be considered a microbial biomarker of a healthy VM (17, 18). L. crispatus appears to be involved in maintaining the homeostasis of the vaginal environment, where it supports the vaginal immune barriers without causing inflammation while at the same time reducing proinflammatory cytokines, which typically increase during bacterial vaginosis (19, 20). Moreover, members belonging to this bacterial species produce various antimicrobial compounds (21, 22). The production of bacteriocins by lactic acid bacteria has been reported for several decades (23), and only recently the genetic determinants of bacteriocins produced by L. crispatus (LCB) have been characterized by *in silico* studies (24, 25). In the context of the latter studies, a total of six putative bacteriocin-encoding genes/loci, named LCB 1 to 6, were predicted to be harbored by eight genomes of L. crispatus strains isolated from vaginal swabs. In addition, two bacteriocin-encoding gene clusters, termed LCB 7 to 8, were found to be located on the genomes of seven L. crispatus strains, which had been isolated from chicken fecal samples (24).

Another powerful antimicrobial compound produced by certain VM-associated lactobacilli (i.e., L. crispatus and L. jensenii) is hydrogen peroxide (26), which is believed to counteract colonization of pathogenic bacteria, thereby exerting a protective role against bacterial infections of the human vagina. However, very little is known about the production of hydrogen peroxide in L. crispatus (27–29) and its potential role as a modulator of the VM. Lactic acid is another metabolic end product produced by L. crispatus with antimicrobial activity. Noticeably, L. crispatus represents a so-called homofermentative lactic acid bacterium, meaning that it is able to degrade sugars through the glycolytic pathway, leading to the formation of pyruvate. The latter is subsequently reduced to lactic acid thanks to the presence of lactate dehydrogenase (LDH) (30). Moreover, previous studies have demonstrated that vaginal L. crispatus strains encode pullulanase activity, which is responsible for the degradation of glycogen, the main carbon source present in the vaginal lumen (31, 32). This property is believed to positively affect the abundance of lactobacilli, in particular L. crispatus, as well as the overall composition of the vaginal microbiota (33).

In the current work, we describe the evaluation of the intraspecies biodiversity of L. crispatus by *in silico* investigations of metagenomic data sets followed by assessment of *in vitro* modulatory effects of L. crispatus strains and other key members of the *Lactobacillus* genus toward the overall vaginal microbiota with a focus on their antibacterial activities against various pathogenic microorganisms associated with vaginal infections (28, 29, 34).

## RESULTS AND DISCUSSION

### *In silico* investigation of the vaginal microbiota and *Lactobacillus* intraspecies biodiversity in metagenomic data sets.

The microbial biodiversity of the vaginal microbiota and the intraspecies biodiversity of the most prevalent *Lactobacillus* taxa typically found in the vaginal environment (i.e., L. crispatus, L. jensenii, *L. iners*, and L. gasseri) were investigated through *k*-mer profile-based analysis of metagenomic data sets obtained from healthy human vaginal microbiota samples. Specifically, we screened publicly available metagenomic data corresponding to the main recognized VCSTs. For each VCST, which is dominated by one of four above-mentioned microbial species (10, 11, 18), 20 data sets (Table S1 in the supplemental material) were processed by reconstruction of species-level taxonomic profiles and analysis of the intraspecies biodiversity of dominating lactobacilli by comparison of *k*-mer profiles with that of a nonredundant database of microbial genomes. The latter was obtained by clustering of all publicly available microbial genomes of L. crispatus, L. jensenii, *L. iners*, and L. gasseri followed by exclusion of genomes with a high Jaccard similarity coefficient (Fig. S1).

Data retrieved revealed that dominance of L. crispatus is associated with lower biodiversity expressed as species richness (average of 2 species) than L. jensenii, *L. iners*, and L. gasseri (average species richness of 6.8, 7.1, and 11, respectively; Fig. S1). Moreover, L. crispatus, when present, is generally represented by a single dominant strain, with an average of 1.13 strains per biological sample of their corresponding VCSTs, while L. jensenii, *L. iners*, and L. gasseri showed an average of 1.75, 2.1, and 1.7 strains. These differences in species and strain biodiversity observed for the four investigated *Lactobacillus* taxa suggest that L. crispatus is characterized by a more pronounced level of interspecies competition for niche colonization than other vaginal lactobacilli. These results are in accordance with recent scientific literature showing metabolic variability among L. crispatus strains, including production and/or degradation of biogenic amines, which can raise vaginal pH and favor opportunistic pathogen colonization (35), and phenotypic responses to host molecules such as estrogens (36). Genes commonly involved in the biogenic amine production pathway are the enzyme arginine decarboxylase (EC 4.1.1.19), which converts arginine into putrescine, lysine decarboxylase (EC 4.1.1.18), which decarboxylates lysine into cadaverine, and tyrosine decarboxylase (EC 4.1.1.25), which converts tyrosine into tyramine (35). We performed a BLAST homology search using a custom database encompassing all known arginine, lysine, and tyrosine decarboxylase sequences available in public databases, which showed that the genes encoding arginine and lysine decarboxylases are conserved among all L. crispatus strains, whereas the gene encoding tyrosine decarboxylase appears to be absent (Table S8). Moreover, almost all poultry strains and just one human strain (i.e., LB61) possess two lysine decarboxylase-encoding genes, a characteristic therefore that seems to be linked to the ecological niche.

Based on these findings, we decided to gain further insight into strain-level L. crispatus variability in terms of their interaction with other vaginal commensals and (opportunistic) pathogens.

### *In vitro* evaluation of antibacterial activities of different strains of L. crispatus.

In order to evaluate the antimicrobial activities of various members of the L. crispatus species against vaginal pathogens, the agar spot diffusion method was used. Fifteen L. crispatus strains isolated from human vaginal swabs or chicken fecal samples (27) were tested against 11 pathogens commonly involved in vaginal infections, such as bacterial vaginosis, vaginal candidiasis, and urinary tract infections (Table 1), also including species that may have originated from fecal contamination (28, 29, 34). Furthermore, additional vaginal *Lactobacillus* species (i.e., L. jensenii, *L. iners*, and L. gasseri) were included in these analyses (Table 1). We decided to evaluate the antimicrobial activity of L. crispatus strains isolated from healthy vaginal swabs as well as from chicken fecal samples based on the high prevalence of this species in these environments in order to evaluate any phenotypic differences linked to the ecological origin (28, 37, 38).

**TABLE 1:** Bacteria used in this study

Species	Strain^*a*^^,^^*b*^	Origin	Accession no. of bacterial strains used in genomic analyses^*c*^
L. crispatus	PRL2021	Vaginal swab	CP058996
L. crispatus	LB56	Vaginal swab	JACCPX000000000
L. crispatus	LB57	Vaginal swab	JACCPW000000000
L. crispatus	LB58	Vaginal swab	JACCPV000000000
L. crispatus	LB59	Vaginal swab	JACCPU000000000
L. crispatus	LB61	Vaginal swab	JACCPT000000000
L. crispatus	LB62	Vaginal swab	JACCPS000000000
L. crispatus	LB63	Vaginal swab	JACCPR000000000
L. crispatus	LB64	Chicken feces	JACCPQ000000000
L. crispatus	LB65	Chicken feces	JACCPP000000000
L. crispatus	LB66	Chicken feces	JACCPO000000000
L. crispatus	LB67	Chicken feces	JACCPN000000000
L. crispatus	LB68	Chicken feces	JACCPM000000000
L. crispatus	LB69	Chicken feces	JACCPL000000000
L. crispatus	LB70	Chicken feces	JACCPK000000000
L. gasseri	V105C	Vaginal swab	–
L. gasseri	ATCC 9857	ATCC collection	–
L. jensenii	V79H	Vaginal swab	–
L. jensenii	V94G	Vaginal swab	–
*L. iners*	LMG 14328	LMG collection	–
Enterococcus faecalis	ATCC 19433	ATCC collection	–
Staphylococcus aureus	ATCC 43300	ATCC collection	–
Staphylococcus epidermidis	ATCC 35984	ATCC collection	–
Streptococcus agalactiae	ATCC 13813	ATCC collection	–
Escherichia coli	ATCC 11775	ATCC collection	–
Klebsiella pneumoniae	ATCC 13883	ATCC collection	–
Pseudomonas aeruginosa	ATCC 27853	ATCC collection	–
Gardnerella vaginalis	LMG 07832	LMG collection	–
Mobiluncus curtisii	LMG 07856	LMG collection	–
Prevotella bivia	LMG 06452	LMG collection	–
Candida albicans	ATCC 32032	ATCC collection	–
B. longum subsp. *longum*	ATCC 15707	ATCC collection	–
B. adolescentis	ATCC 15703	ATCC collection	–
*L. paracasei* ATCC 334	ATCC 334	ATCC collection	–
Bacteroides ovatus	PR2	Infant fecal sample	–
Escherichia coli	Nissle 1917	Probiotic product	–

a ATCC, American Type Culture Collection; LMG, Belgian Coordinated Collections of Microorganisms.

b V105C, ATCC 9857, V79H, V94G, LMG 14328, ATCC 19433, ATCC 43300, ATCC 35984, ATCC 13813, ATCC 11775, ATCC 13883, ATCC 27853, LMG 07832, LMG 07856, LMG 06452, ATCC 32032, ATCC 15707, ATCC 15703, ATCC 334, PR2, and Nissle 1917 were not sequenced, because they were used only in the physiological experiments and not the genetic experiments.

c –, no accession number.

**FIG 1:**
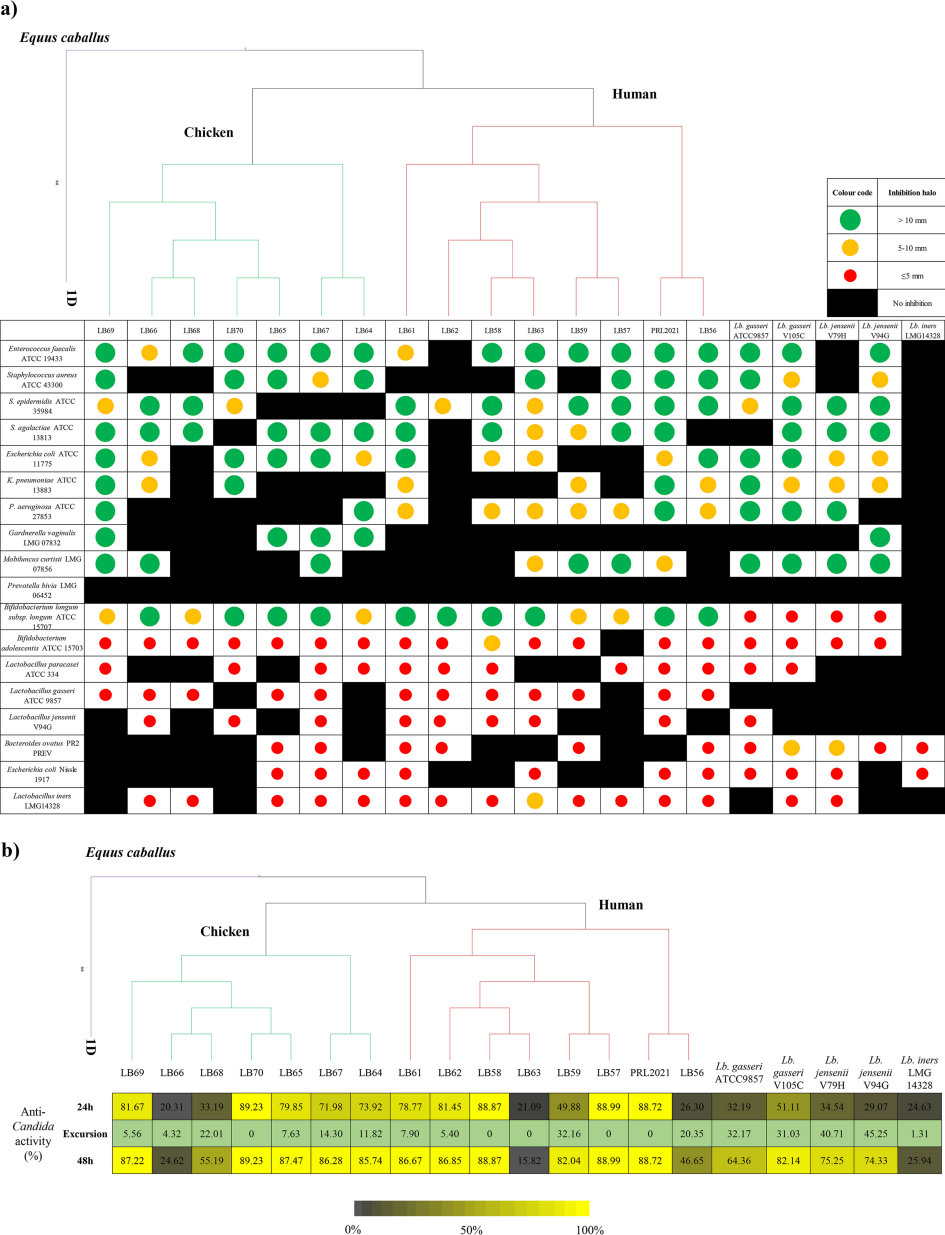
Antibacterial activity of *Lactobacillus* strains against different pathogens. (a) A phylogenetic tree of 17 Lactobacillus crispatus strains, including eight isolated from humans, eight from chicken, and one from Equus caballus, and its association with the diameter of the inhibition halos obtained for each *Lactobacillus* species grown with various (opportunistic) pathogens. (b) A phylogenetic tree of 17 Lactobacillus crispatus strains, including eight isolated from humans, eight from chicken, and one from Equus caballus, and its association with antimicrobial activity of *Lactobacillus* CFS toward Candida albicans ATCC 32032, tested following EUCAST guidelines. OD_530 nm_ values at 24 h and 48 h were normalized for positive controls, and results are expressed as inhibition (%) of *Candida* growth. Excursion represents the difference between the inhibition (%) between 48 h and 24 h.

All tested *Lactobacillus* species, with the exception of *L. iners*, were shown to elicit antagonistic activity against the majority of pathogenic strains assayed, including opportunistic pathogens of intestinal origin. Notably, while species-specific differences were observed as expected, we also observed marked strain-specific differences among the L. crispatus strains in terms of inhibitory activity and inhibitory spectrum (Fig. 1; Table 2). Such results do not directly correlate with the phylogenetic relationship of these strains (Fig. 1). For example, Staphylococcus epidermidis is inhibited specifically by L. crispatus isolates of human origin, while Gardnerella vaginalis is inhibited exclusively by assessed strains of poultry origin, and Enterococcus faecalis is inhibited by the majority of tested L. crispatus strains from either of these ecological niches (Fig. 1; Table 2).

**TABLE 2:** Antibacterial activity of *Lactobacillus* strains against different pathogens

*Lactobacillus* strains	Firmicutes^*a*^				Proteobacteria^*a*^			Actinobacteria^*a*^		Bacteroidetes^*a*^	Fungi^*a*^
	Enterococcus faecalis ATCC 19433	Staphylococcus aureus ATCC 43300	Staphylococcus epidermidis ATCC 35984	Streptococcus agalactiae ATCC 13813	Escherichia coli ATCC 11775	Klebsiella pneumoniae ATCC 13883	Pseudomonas aeruginosa ATCC 27853	Gardnerella vaginalis LMG 07832	Mobiluncus curtisii LMG 07856	Prevotella bivia LMG 06452	Candida albicans ATCC 32032
PRL2021	13	12	12	13	10	19	12	0	10	0	0
LB56	19	12	12	0	12	10	10	0	0	0	0
LB57	12	11	17	11	0	0	10	0	13	0	0
LB58	14	0	23	12	10	0	10	0	0	0	0
LB59	13	0	19	10	0	10	7	0	15	0	0
LB61	10	0	20	11	11	10	10	0	0	0	0
LB62	0	0	10	0	0	0	0	0	0	0	0
LB63	20	19	8	9	9	0	6	0	10	0	0
LB64	15	16	0	11	9	0	13	12	0	0	0
LB65	20	14	0	12	12	0	0	12	0	0	0
LB66	10	0	20	13	9	10	0	0	14	0	0
LB67	11	9	0	19	11	0	0	12	13	0	0
LB68	13	0	13	11	0	0	0	0	0	0	0
LB69	16	13	10	24	12	11	17	12	12	0	0
LB70	18	14	10	0	13	11	0	0	0	0	0
L. gasseri ATCC 9857	16	12	10	0	12	11	13	0	16	0	0
L. gasseri V105C	16	10	11	12	12	10	12	0	13	0	0
L. jensenii V79H	0	0	11	31	10	10	15	0	12	0	0
L. jensenii V94G	16	10	11	13	10	8	0	12	14	0	0
*L. iners* LMG14328	0	0	0	0	0	0	0	0	0	0	0

a Values are the mean of duplicate measurements of inhibition halo (mm).

Noticeably, among the eight *Lactobacillus* strains isolated from vaginal samples, the L. crispatus PRL2021 strain was shown to be the most effective in inhibiting the growth of tested pathogens, being capable of antagonizing 8 out of 11 tested pathogens (Fig. 1; Table 2), together with the poultry isolate L. crispatus LB69 that was shown to inhibit the growth of 9 out of 11 tested pathogens. With the aim to evaluate anti-*Candida* activity, we performed the microdilution method, following the EUCAST guidelines (*Materials and Methods*). Overall, cell-free supernatants (CFS) from most L. crispatus strains exhibit high levels of *Candida* growth inhibition (over 80%; Fig. 1), except for five strains (i.e., LB56, LB59, LB63, LB66, and LB68), which showed a distinctly lower level of antifungal activity (under 50%; Fig. 1). These findings clearly confirm strain-specific differences of the L. crispatus species. In contrast, L. gasseri, L. jensenii, and *L. iners* strains showed a comparatively low level of anti-*Candida* activity, which ranged from 20% to 50% (Fig. 1). These results suggest that lactobacilli display antimicrobial activities that are strain and species specific, although not correlated with the phylogenetic tree. This may be the result of evolutionary pressure in terms of competition for niche colonization.

In contrast, the inhibitory activities exerted by L. crispatus strains against other commensal *Lactobacillus* species was limited to a small number of strains, and, when such activity was noted, it was presented as a small inhibition halo (Fig. 1; Table 3). In this regard, L. crispatus LB63 showed stronger antibacterial activity against *L. iners* LMG 14328, displaying a halo with a diameter of 7.5 mm (Fig. 1; Table 3). This might be explained by the fact that L. gasseri, L. jensenii, *L. paracasei*, and *L. iners* strains naturally colonize the human vaginal tract alongside L. crispatus and thus may have adopted a cohabitation strategy rather than promoting competitive behavior (11).

**TABLE 3:** Antibacterial activity of *Lactobacillus* strains against different bacterial species

*Lactobacillus* strains	Actinobacteria^*a*^^,^^*b*^		Firmicutes^*a*^^,^^*b*^				Bacteroidetes^*a*^^,^^*b*^	Proteobacteria^*a*^^,^^*b*^
	Bifidobacterium longum *subsp. longum* ATCC 15707	Bifidobacterium adolescentis ATCC 15703	Lactobacillus paracasei ATCC 334	Lactobacillus gasseri ATCC 9857	Lactobacillus jensenii V94G	Lactobacillus iners LMG 14328	Bacteroides ovatus PR2	Escherichia coli Nissle 1917
PRL2021	14	4	2.5	3.5	1	1	0	1.5
LB56	12.5	4.5	3	3	0	1.5	1	1
LB57	5.5	0	2	–	0	2.5	0	0
LB58	15	7	2.5	3.5	1	3.5	0	0
LB59	6	3	0	1	0	4	1	0
LB61	14.5	3.5	2.5	3.5	1.5	7	1	1
LB62	14.5	4.5	1.5	2.5	2	3	1	0
LB63	10.5	3.5	0	2	1.5	7.5	0	1
LB64	8	3.5	1	–	0	3	0	1.5
LB65	11	5	0	2.5	0	4.5	2	2.5
LB66	10.5	4	0	2.5	1	4	0	0
LB67	16	4	1	5.5	2	4	1	3.5
LB68	7.5	3	0	5	0	0	0	0
LB69	6	4.5	1	2	0	0	0	0
LB70	17	4.5	1.5	4	1.5	0	0	0
L. gasseri ATCC 9857	1	1	1	–	1	3	5	1
L. gasseri V105C	1.5	3.5	2	–	0	1	7.5	1
L. jensenii V79H	2	3	0	0	–	0	6.5	2
L. jensenii V94G	1	3	0	0	–	3	3.5	0
*L. iners* LMG14328	0	0	0	0	0	–	1	1

a Values are the mean of duplicate measurements of inhibition halo (mm).

b –, no data.

Altogether, the inhibition assays highlight a marked strain-level diversity among the tested lactobacilli in terms of their ability to inhibit growth of other (opportunistic) colonizers of the vaginal environment.

Based on these phenotypic data, we decided to investigate the mechanism(s) responsible for the observed antibacterial activities of L. crispatus strains.

### Bacteriocin prediction and pathogen inhibition.

**FIG 2:**
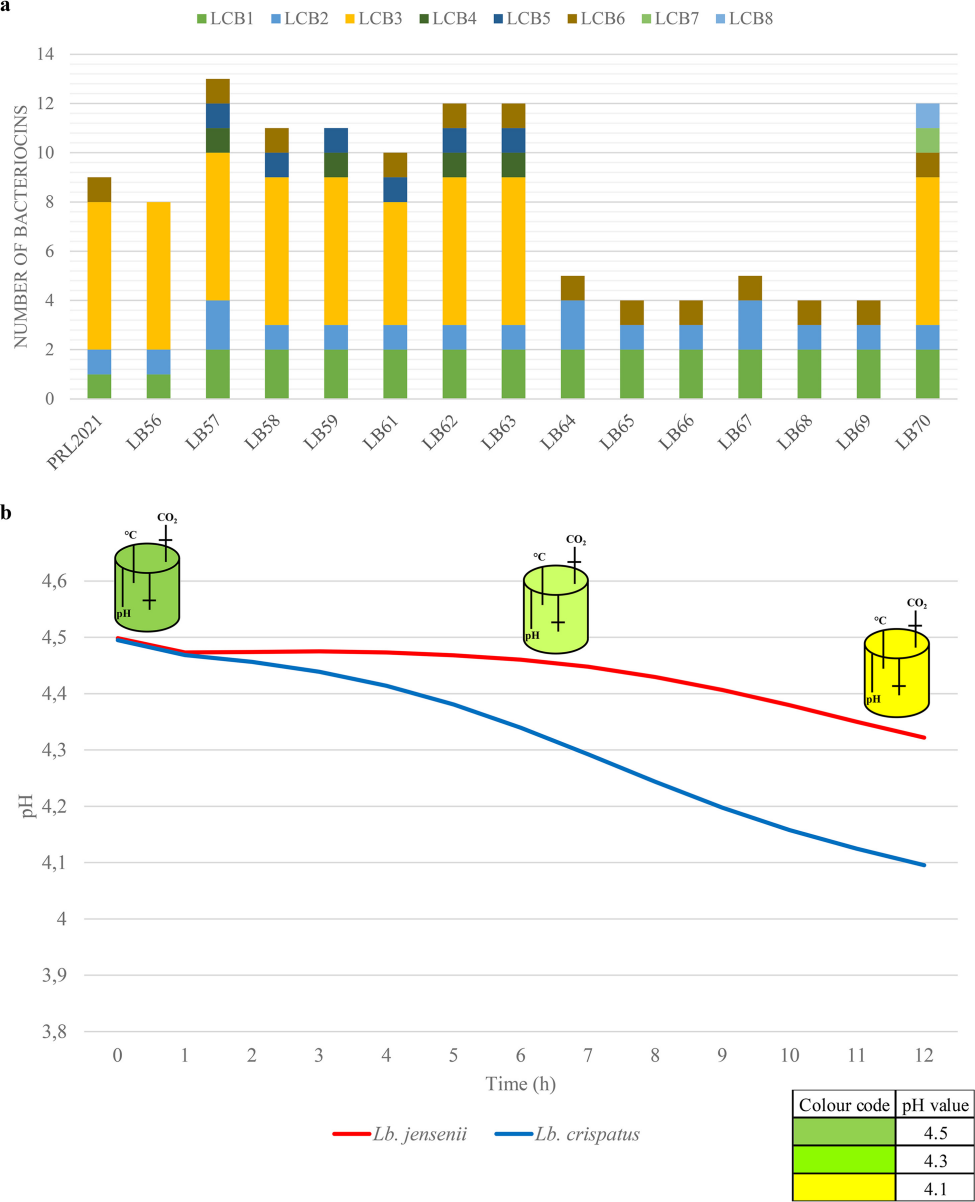
Evaluation of antimicrobial compounds in L. crispatus strains. (a) A graphical count of bacteriocin-associated loci identified in L. crispatus and represented as a bar plot. (b) pH decrease (due to lactic acid production) during fermentation in simulated vaginal fluid (SVF). The variation of pH is reported as a function of time.

Bacteriocins are antimicrobial peptides produced by bacteria that are released into the surrounding environment in order to enhance competitiveness toward other microorganisms for niche colonization (39). Screening for the presence of genes associated with bacteriocin production in the genomes of L. crispatus species allowed the identification of eight putative bacteriocin genes/loci (LCBs) distributed across L. crispatus strains of both human and poultry origin, with the only exception of LCBs 4 and 5, which were exclusively observed in isolates from human hosts, and LCBs 7 and 8, which were only identified in a strain of poultry origin, as previously reported (24, 40) (Fig. 2; Table S2).

The presence of genes associated with bacteriocin production was statistically correlated with halo sizes obtained from *in vitro* experiments of antimicrobial activity (Fig. 1; Table S2). However, due to a heterogeneous distribution of LCBs among analyzed strains, only LCBs 3, 4, and 5 could be included in the *t* test statistical analysis (Fig. S2; Table S2). In fact, LCBs 1, 2, and 6 are widespread among all analyzed strains, suggesting that these may have an ecological relevance for all members of the L. crispatus species. Furthermore, LCBs 7 and 8 were excluded since they were present in the chromosome of just a single poultry strain, therefore not allowing statistical investigations.

The antimicrobial data revealed that the presence of LCBs 3 and 5 is significantly correlated with a higher average diameter of the inhibition halo for Staphylococcus epidermidis (Fig. S2; Table S2), thus suggesting a possible target for the corresponding bacteriocins. Intriguingly, we also observed negative correlations, such as for LCBs 3 and 5, both associated with a reduced inhibition halo of Gardnerella vaginalis. Furthermore, LCB 3 was negatively correlated with Streptococcus agalactiae inhibition, while LCB 4 was negatively correlated with Escherichia coli. Interestingly, LCB 3 is comprised of six genes that are predicted to encode a small peptide pheromone along with a bacteriocin immunity protein, normally transcribed together with the bacteriocin in order to protect the producer bacteria (41), a putative ABC transporter, and a two-component regulatory system (24). Bioinformatic analysis suggests that this locus corresponds to the inhibition activity against Streptococcus species (41). Moreover, this locus is absent in L. crispatus species isolated from poultry, while it is found in all isolates from vaginal samples. In contrast, LCB 5 is widespread among various lactic acid bacteria (i.e., *Lactobacillus* spp., *Enterococcus* spp., *Pediococcus* spp., *Leuconostoc* spp., and *Carnobacterium* spp.) (24, 42–44).

**FIG 3:**
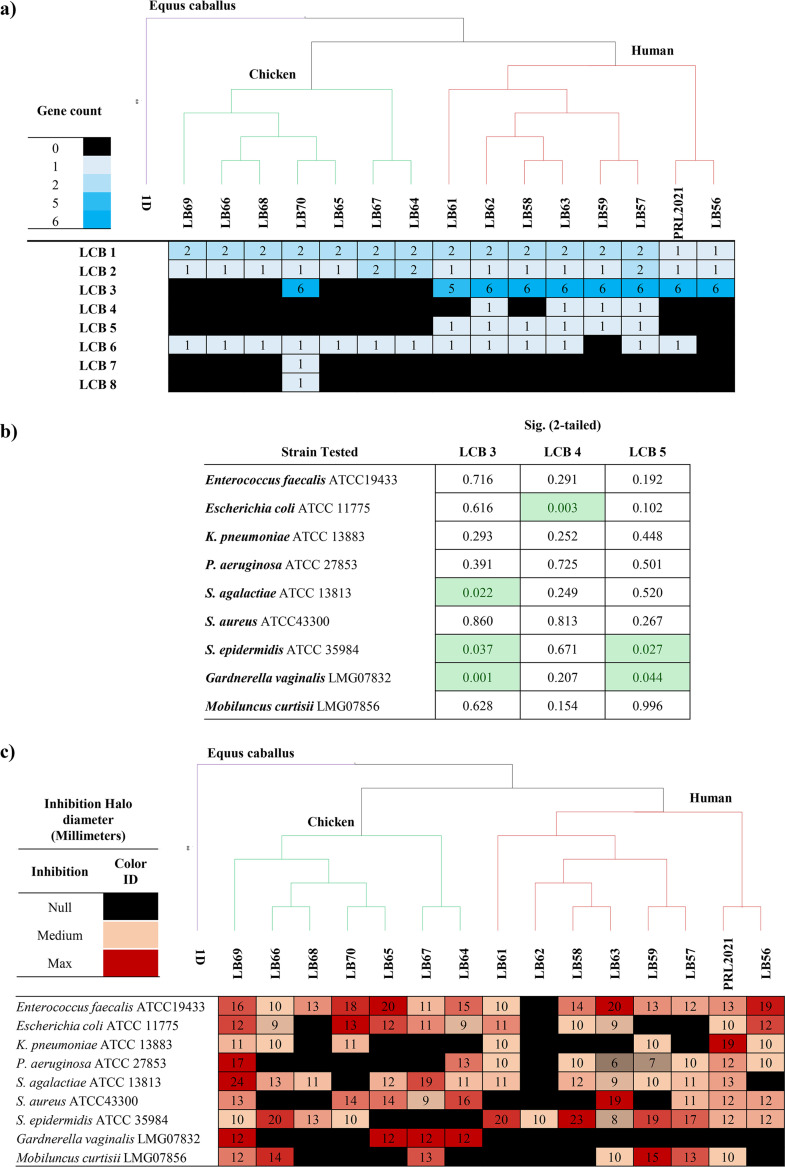
Phylogenetic tree of L. crispatus and related inhibition data. (a and c) A phylogenetic tree of 17 Lactobacillus crispatus strains, eight isolated from human, eight from chicken, and one from Equus caballus, is related to bacteriocin distribution with relative gene count (a) and to inhibition halo, expressed in millimeters, of nine different species used as a control test (c). (b) Statistical analysis relative to impact of bacteriocin LCBs 3, 4, and 5 versus the nine species tested.

While these results do not provide definite indications regarding the microbial targets of such bacteriocins, probably also due to the confounding effect of media acidification, these data do suggest a link between the phylogenetic relationship between strains and their ability to inhibit other bacterial taxa. Thus, we tested this notion by linking halo sizes and the presence/absence of bacteriocin families with a core gene-based phylogenetic positioning of the investigated strains (Fig. 3; Fig. S2). A comparative genomic analysis was performed using 1,034 identified L. crispatus core genes based on the strains assessed in this study to investigate the phylogenetic relationship. Notably, this approach for phylogenetic reconstruction, based on alignment of all the genes shared by a set of genomes, has been proven to provide high resolution, as described previously (45, 46). Intriguingly, while the presence of bacteriocin families correlates with the phylogenetic clusters observed in the core gene supertree, the latter does not correlate with the inhibition halo data. Remarkably, these observations support the notion that vaginal lactobacilli still undergo significant evolutionary development to improve their competitiveness with cocolonizer species, which cannot be directly correlated with acquisition or loss of genes, thus suggesting a role of divergent evolution of specific genes in terms of sequence and expression. No significant correlation was observed between the quantity of lactic acid and halo sizes obtained from *in vitro* experiments of each antimicrobial activity test (Fig. 1).

### Evaluation of lactic acid production by vaginal lactobacilli.

It is widely accepted that the production of organic acids, especially lactic acid, contributes to the lowering of the vaginal pH and therefore making this environment unfavorable for growth of pathogenic microorganisms (47). In order to evaluate the metabolic performances of the lactobacilli isolated from the vaginal tract in terms of lactic acid production, we applied two distinct experimental sets, both involving a bioreactor model inoculated by i) a synthetic growth medium (i.e., De Man Rogosa and Sharpe [MRS] medium) or ii) a medium simulating the vaginal fluid (simulated vaginal fluid [SVF]) (48).

During the first 3 h, the pH remained constant for all *Lactobacillus* species tested (Fig. 2; Fig. S3 and Table S3), after which it rapidly decreased to 4.0. In this context, *L. iners* lowered the pH to 4.0 in about 20 h (Fig. 2; Fig. S3 and Table S3), thus representing the species with the slowest acidification times, while the two L. jensenii strains caused acidification of the medium to pH 4.0 in just 4 h, thus being the fastest acidifiers. Notably L. jensenii, unlike the other *Lactobacillus* species tested, is a facultative heterofermentative; therefore, its fast acidification may be due to the formation of other types of acids besides lactic acid (49). Remarkably, the L. crispatus strains tested showed variable acidification times that ranged from 5 h to 9 h (Fig. 2; Fig. S3 and Table S3). Moreover, for all tested *Lactobacillus* strains, the steady-state condition was reached after around 13 h of incubation with a pH value of 3.8, at which point cells are no longer able to grow, probably due to the high toxicity of lactic acid (plateau phase, optical density at 600 nm [OD_600 nm_] ∼ 3.0) (50) (Fig. S3; Table S3). Although lactobacilli are known to survive well under low pH conditions, we observed that each tested strain showed a specific performance with regard to its acidification rate, perceptible also among L. crispatus species, a feature that has been previously correlated with increased ecological fitness and colonization performance (Fig. 2; Fig. S3 and Table S3) (51–53). All tested strains produced lactic acid in the range of 5 mM to 8.82 mM (Table 4; Table S4).

**TABLE 4:** Production of lactic acid in cultures of lactobacilli

		Concn lactic acid (mM)/10^8^ cells	
Species	No. of strains	Mean	SD
L. crispatus	8	8.30	0.52
L. gasseri	2	7.90	0.03
L. jensenii	2	8.51	0.08
*L. iners*	1	5.06	3.58

The more powerful pH-lowering capabilities, lactic acid production, higher number of bacteriocin clusters, and larger halos of L. crispatus and L. jensenii than L. gasseri and *L. iners* as observed in MRS (Table 4; Table S4) were also validated through fermentation of one strain per species in SVF. Interestingly, the tested lactobacilli were shown to cause a pH decrease from 4.5 to 4.0 in 12 h except for L. jensenii V94G, which reduced the pH to 4.0 in 24 h (Fig. S3; Table S3). Interestingly, fermentation on SVF resulted in an even stronger pH-lowering performance of L. crispatus than fermentation on MRS, highlighting their superior pH-lowering ability compared to L. jensenii (Fig. 2). These findings suggest that L. crispatus has adapted specifically to the vaginal environment, which could give this species greater ecological fitness in this specific ecological niche.

### Genomic investigation of lactate-producing genes.

Production of lactic acid, present as l- and d-isomers, is considered the main reason for pH reduction by homofermentative *Lactobacillus* species (54). Thus, in order to investigate the genetic basis for the various acidification performances observed for L. crispatus strains included in this study, we performed a BLAST homology search using a custom database encompassing all known lactate dehydrogenase sequences available in public databases. Results showed that all L. crispatus strains isolated from the human vagina possess two l-lactate dehydrogenase-encoding genes (DH_1 and DH_2) and a single d-lactate dehydrogenase-encoding gene (DH_3), showing therefore, the same genetic repertoire for the production of d/l-lactic acid.

Among the eight analyzed strains of human-derived L. crispatus, we found that the DNA sequences of the three lactate dehydrogenase-encoding genes are highly conserved, with the presence of only five single-nucleotide polymorphisms (SNPs) in DH_1 and only one SNP in DH_3 (Fig. S4). Additionally, only one SNP present in DH_1 was a nonsynonymous SNP affecting the protein sequence. However, no correlation was found between SNP and pH data, indicating that this mutation is not relevant to the functionality of the encoded protein (Fig. S4). This may therefore be an indication of the key biological relevance of these three genes for L. crispatus strains. Nevertheless, future investigation of their transcriptional profiles may provide additional clues regarding the different performances of acidification rates observed for the investigated L. crispatus strains (Table S4).

### Coculture experiments to evaluate competitive behavior on glycogen.

In order to define the cutoff value of glycogen as a growth-limiting factor, we evaluated growth performances of the different *Lactobacillus* strains used in coculture experiments on different concentrations of glycogen. We observed that each strain has different needs, ranging from 0.25% to 2% (OD > 0.5); therefore, we decided to use a moderate level of 0.5% glycogen in the coculture experiments (Table S5). In this context, results confirmed that L. crispatus is a species that is well adapted to growth on glycogen, since all tested strains are able to exhibit superior growth on this carbon source (OD > 0.5) than other vaginal lactobacilli, such as L. jensenii and L. gasseri, thus confirming previous studies (27, 32) (Table S5). These findings may explain the higher colonization and acidification performances of the L. crispatus species in the vaginal environment since glycogen is a complex carbohydrate commonly present in human vaginal fluid (32). To evaluate glycogen breakdown capabilities of the various *Lactobacillus* species that colonize the human vagina, we decided to perform nine different cocultivation experiments where two strains of L. crispatus (i.e., PRL2021 and LB57) were coinoculated with other representative microorganisms of the VM (i.e., L. jensenii, *L. iners*, L. gasseri, and *G. vaginalis*) with glycogen as the sole carbon source. Strains PRL2021 and LB57 were selected as representatives of the L. crispatus species since they showed antibacterial activity against different pathogens (Table 2), while they also appear to effectively decrease the pH of the simulated vaginal fluid (Fig. S3). A quantitative PCR (qPCR) approach was used to quantify the bacterial DNA of each species relative to the total DNA extracted from coculture cultivation experiments.

**FIG 4:**
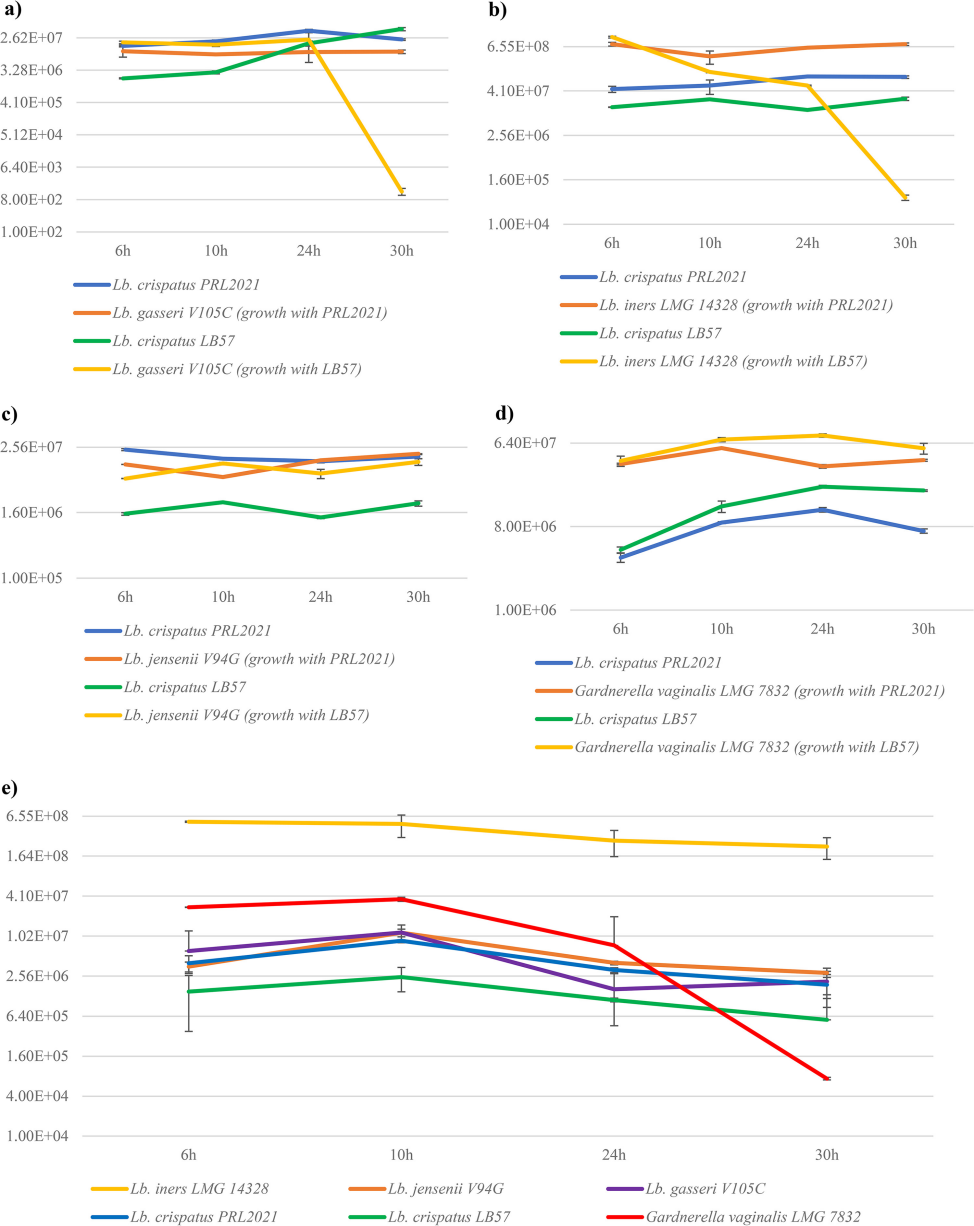
Evaluation of *Lactobacillus* load in coculture experiments. Quantitative PCR evaluation of the relative number of *Lactobacillus* and *Gardnerella* strains in coculture experiments. The graph highlights the average abundance observed through qPCR at 6 h, 10 h, 24 h, and 30 h. (a) Coculture results of two different experiments in which PRL2021 or LB57 L. crispatus strains were grown together with L. gasseri V105C. (b) Relative load of L. crispatus strains (PRL2021 or LB57) with *L. iners* LMG 14328. (c) Relative number of L. crispatus strains (PRL2021 or LB57) with L. jensenii V94G. (d) qPCR evaluation between L. crispatus strains (PRL2021 or LB57) and Gardnerella vaginalis LMG 7832. (e) Relative load of all microorganisms used in the cocultivation experiments when cocultivated together.

The qPCR analysis revealed diverging performances of the two L. crispatus strains. In detail, L. crispatus PRL2021 appears to grow well in coculture with L. gasseri V105C or *L. iners* LMG 14328 showing no sign of competitive exclusion or antagonistic behavior, whereas L. crispatus LB57 seemed to strongly compete with or antagonize growth of the latter species (Fig. 4). However, both L. crispatus strains were shown to grow together with L. jensenii V94G without noticeable effect on their growth behavior (Fig. 4). As mentioned above, these data confirm that even phylogenetically related L. crispatus strains isolated from the same ecological niche display diverging phenotypes that may be associated with ecological fitness and competition with other microbial colonizers (55). In the case of the coculture between *G. vaginalis* LMG 7832 and vaginal lactobacilli used in this experiment, we observed that the included *Lactobacillus* species are able to cohabit without apparent competition (Fig. 4). This finding may be explained by the fact that these bacteria share the same ecological niche and thus may have evolved cohabitation strategies, corroborated by the observation that L. gasseri and L. jensenii struggle to grow on a medium containing glycogen as the sole carbon source. Instead, in cocultures with L. crispatus, they exhibited increased growth (Fig. 4), indicative of cross-feeding behavior. This possibility seems to be confirmed by the glycogen consumption pattern, as in cocultures after 30 h of growth, glycogen was completely consumed in contrast to the monoassociations. It could be assumed that L. crispatus metabolizes glycogen early, providing simpler structures for the growth of other *Lactobacillus* species (Table S6). These data corroborate the notion that all *Lactobacillus* species typically found in the vaginal environment are genetically adapted to grow on glycogen as the main shared carbon source. The glycogen breakdown capabilities of L. crispatus strains used (i.e., LB57 and PRL2021) is further confirmed by the presence of a conserved gene cluster (Fig. S5). This conserved set of genes encompasses a gene encoding a predicted amylase (GH13) followed by genes that are predicted to specify a maltose phosphorylase, a β-phosphoglucomutase, and an ABC (ATP-binding cassette) system for carbohydrate uptake (Fig. S5), all of which are implicated in glycogen degradation (33). The specific adaptation of these *Lactobacillus* species to the vaginal environment is also confirmed by the observation that the cell number of *G. vaginalis* appears to decrease over time when various *Lactobacillus* species typical of the VM are cocultivated together, underscoring the ability of lactobacilli toward collaborative utilization of this carbon source and concomitant inhibition of other microbial taxa. Future studies will therefore involve cocultivation experiments between the two *L. crispatus* representatives (i.e., PLR2021 and LB57) in order to perform an in-depth evaluation of possible competition mechanisms between them in the complex vaginal environment.

### Conclusions.

The human VM includes all microorganisms that colonize the vaginal tract. Among these, L. crispatus is reported to exert a key role in maintaining host health. In the current study, we performed *in vitro* experiments in order to evaluate the possible modulatory effects of L. crispatus strains by maintaining the host VM homeostasis through the production of different antimicrobial compounds, such as bacteriocins and lactic acid. Furthermore, the ability of this species to interact with other members of this ecological niche was evaluated in carbohydrate competition experiments.

Our findings revealed that lactobacilli typically found in the vaginal environment appeared to have adapted to this niche and perhaps are still undergoing adaptation in terms of improved competition for ecological niche colonization. From a species-level perspective, L. crispatus showed the most advanced colonization performance, while various strain-specific characteristics were observed even in phylogenetically closely related strains (i.e., differences in gene content and sequence divergence) as well as phenotypic characteristics. Particularly, in this work, we highlighted some intriguing novel aspects about high genetic variability of L. crispatus in terms of antibacterial activity against human vaginal pathogens as well as the production of bacteriocins and other antimicrobial compounds such as lactic acid. All together, these findings clearly support the notion of L. crispatus as an important driver of the vaginal microbiota. In this regard, further investigations encompassing a large collection of L. crispatus strains and additional multiomics approaches will be pivotal to elucidate the evolutionary mechanisms that drive adaptation to the vaginal environment.

## MATERIALS AND METHODS

### Taxonomic profiling and strain tracking.

We selected 80 different vaginal samples from four different BioProject numbers (PRJNA48479, PRJNA275349, PRJNA576566, and PRJEB38528; Table S1 in the supplemental material) aimed at analyzing the taxonomic profiles. METAnnotatorX2 (56) was used with default settings, 50,000 input reads, and human read filtering. We retrieved from the public data set a representative complete subset of data sequenced on an Illumina MiSeq platform (57). The strain tracking analysis was instead carried out using all publicly available genomes of L. crispatus, *L. iners*, L. gasseri, and L. jensenii through the StrainGE software (https://github.com/broadinstitute/StrainGE), with default parameters, against 80 publicly available vaginal shotgun sequencing samples (selected based on its high-depth sequencing) and downloaded from the Sequence Reads Archive (SRA) of NCBI (Table S1).

### Strains and culture conditions.

*Lactobacillus* strains used in this study are shown in Table 1 (27). The strains used were isolated as part of previous work in which written informed consent from each donor was obtained prior to inclusion in the study. Lactobacilli were grown anaerobically in De Man, Rogosa, Sharpe (MRS) medium (Scharlau) supplemented with 0.05% l-cysteine-HCl and incubated at 37°C for 24 h. Anaerobic conditions were achieved by the use of an anaerobic cabinet (Ruskin), in which the atmosphere consisted of 17% CO_2_, 80% N_2_, and 2.99% H_2_. Pathogenic microorganisms and other microorganisms used in this study are listed in Table 2. Indicator microorganisms for antimicrobial activity were grown aerobically on brain heart infusion (BHI) broth at 37°C for 24 h. The strains Gardnerella vaginalis LMG 07832, Mobiluncus curtisii LMG 7856, and Prevotella bivia LMG 06452 were obtained from Belgian Coordinated Collections of Microorganisms. These microorganisms were cultivated anaerobically on BHI supplemented with 5% of defibrinated horse blood (Thermo Scientific Oxoid) at 37°C for 48 h. Bifidobacteria were grown anaerobically in MRS medium (Scharlau) supplemented with 0.05% l-cysteine-HCl and incubated at 37°C for 24 h. Anaerobic conditions were achieved by the use of an anaerobic cabinet (Ruskin), in which the atmosphere consisted of 17% CO_2_, 80% N_2_, and 2.99% H_2_. Bacteroides ovatus PR 2 was grown anaerobically on BHI broth at 37°C for 24 h. Escherichia coli Nissle 1917 was grown aerobically on Luria-Bertani broth (LB) at 37°C for 24 h.

### Experimental design of the *in vitro* trial.

The antibacterial activity of the selected *Lactobacillus* species isolates was tested by agar spot diffusion test (28, 29, 58, 59) against 13 microorganisms, including both pathogens and commensals (i.e., Enterococcus faecalis ATCC 19433, Staphylococcus aureus ATCC 43300, Staphylococcus epidermidis ATCC 35984, Streptococcus agalactiae ATCC 13813, Escherichia coli ATCC 11775, Klebsiella pneumoniae ATCC 13883, Pseudomonas aeruginosa ATCC 27853, Gardnerella vaginalis LMG 07832, Mobiluncus curtisii LMG 7856, Prevotella bivia LMG 06452, Candida albicans ATCC 3203, Bacteroides ovatus PR2, and Escherichia coli Nissle 1917). These cultures were spread onto BHI agar medium plates. Furthermore, the antibacterial activity of *Lactobacillus* spp. was tested against Bifidobacterium longum subsp. *longum* ATCC 15707, Bifidobacterium adolescentis ATCC 15703, Lactobacillus paracasei ATCC 334, Lactobacillus gasseri ATCC 9857, Lactobacillus jensenii V94G, and Lactobacillus iners LMG 14328. In this case, cultures were spread onto MRS agar medium plates. Specifically, overnight cultures of microorganisms were diluted in order to obtain a final inoculum with an OD_600 nm_ of 1.0 (bacterial cell counts standardized to 2.25 × 10^8^ CFU/mL). The different *Lactobacillus* strains were spotted (10 μL) on the medium containing the competing microorganism (two spots per plate).

Then, plates were incubated for 48 h at 37°C in aerobic or anaerobic atmosphere depending on pathogenic and commensal strains used (see above).

The antimicrobial activity was assessed by the diameter size of the inhibition halo. An inhibition halo with a diameter smaller than 5 mm was considered low inhibition, a halo with a diameter between 5 and 10 mm was perceived as medium inhibition, and a halo with a diameter larger than 10 mm was considered a high level of inhibition, as previously described (29).

### Anti-*Candida* activity of *Lactobacillus* cell-free supernatants.

Anti-*Candida* activity of cell-free supernatants (CFS) was tested by microdilution assay, following EUCAST guidelines (60, 61). For this purpose, lactobacilli were first grown anaerobically in MRS medium supplemented with 0.05% l-cysteine-HCl and incubated at 37°C for 72 h. Cultures were then harvested by centrifugation (2,750 × *g*, 10 min) and filtered through a 0.2-μm filtropur to obtain CFS. CFS obtained were stored at −20°C until their use. Subsequently, stock Candida albicans ATCC 32032 suspensions prepared in sterile water at 0.5 McFarland was diluted appropriately in RPMI 1640 medium (Gibco, Thermo Fisher Scientific, USA) buffered to pH 7.0 with 0.165 M morpholinepropanesulfonic acid buffer (MOPS; Merck, USA) and supplemented with 2% glucose. Final yeast suspensions, corresponding to 10^5^ CFU/mL, were inoculated in flat-bottomed 96-well plates (0.1 mL per well) and added with the same volume of each *Lactobacillus* CFS. Positive growth control wells contained 0.1 mL of *Candida* suspension added with the same volume of sterile MRS medium. The plates were incubated at 37°C for 24 h and 48 h, and *Candida* growth was evaluated by reading the absorbance at 530 nm with a VICTOR Nivo multimode microplate reader. *Candida* growth inhibition was calculated relative to the absorbance of the corresponding positive controls, as previously reported (62).

### Evaluation of lactic acid production by vaginal lactobacilli.

Lactobacilli were grown anaerobically in simulated vaginal fluid (SVF) (48) or in MRS in order to adapt the microorganisms to the medium at 37°C for 24 h. We decided to use these two media because MRS is a synthetic medium of choice for lactobacilli (63), while SVF is a medium that simulates the vaginal environment. Anaerobic conditions were achieved by the use of an anaerobic cabinet (Ruskin), in which the atmosphere consisted of 17% CO_2_, 80% N_2_, and 2.99% H_2_. Revitalized cells at exponential phase (OD_600 nm_ between 0.6 and 0.8) were inoculated under anaerobic conditions in 500 mL of SVF or MRS medium and cultivated in a bioreactor system (Solaris Biotech Solutions, Italy). Strains were cultivated for 24 h at 37°C with mechanical agitation at 180 rpm, and pH variation was recorded every 45 s through a pH meter. At the end of the homofermentative process, the production of lactic acid was measured enzymatically using a d/l-lactic acid (d/l-lactate) (Rapid) assay kit (Megazyme, Bray, Ireland).

### Enzymatic identification of lactic acid.

A d/l-lactic acid (d/l-lactate) (Rapid) assay kit (Megazyme, Bray, Ireland) was used to assess the quantity of each optically active form of lactic acid produced by the fermentation of the microorganism. The assay was performed following the manufacturer’s instructions. Briefly, an aliquot of the fermentation of the single strain was taken for the enzymatic assay. Then, 5 mL of this aliquot was centrifuged at 10,000 rpm for 5 min at 4°C, and the supernatant was filtered through a 0.2-µm filter. Next, 50 µL of the supernatants filtered was submitted for the analysis. Later, the 750 µL of sterile distilled water was added to each supernatant as well as the solutions included in the test kit, such as a buffer (250 µL), NAD^+^ solution (50 µL), and d-GTP (10 µL). After 3 min of incubation, the optical density was measured (OD), and the d-LDH or l-LDH (10 µL) was added and incubated. The collected OD values were used for the calculation of the total quantity of lactic acid following the manufacturer’s instructions.

### Genomic analyses.

With the aim to build a phylogenetic tree on bacteriocin gene production of the different L. crispatus strains, a pangenome calculation was performed using the pangenome analysis pipeline (PGAP), including each L. crispatus genome included in this study (Table S7). Each predicted proteome of a given L. crispatus strain was screened for orthologues against the proteome of every other assessed L. crispatus strain by means of BLAST analysis (cutoff, E value of <1 × 10^−4^ and 50% identity over at least 80% of both protein sequences). The resulting output was then clustered into protein families by means of MCL (graph theory-based Markov clustering algorithm) using the gene family method. A pangenome profile was built using all possible BLAST combinations for each genome being sequentially added. Using this approach, unique protein families encoded by the analyzed L. crispatus genomes were also identified, ranging from 29 for L. crispatus PRL2021 to 164 for L. crispatus LB58. Protein families shared between analyzed genomes allowed us to identify the core genome, which encompasses 1,037 genes, of the L. crispatus species. Each set of orthologous proteins, belonging to the core genome, was aligned using Mafft software, and phylogenetic trees were constructed using ClustalW. Based on these comparative analyses, a L. crispatus supertree was constructed and visualized using FigTree (http://tree.bio.ed.ac.uk/software/figtree/). The presence of potential genes for bacteriocins was evaluated using the Bagel4 software for each single genome of L. crispatus, and all those sequences that turned out to be incomplete hits were removed.

### Statistical analysis.

Statistical analyses such as the independent *t* test were carried out using SPSS software, while covariance and hierarchical clustering (HCL) analyses were carried out using Origin Pro 2021b. Statistical analyses always included the inferential Levene test for the equality of variances.

### Glycogen growth assays.

L. crispatus PRL2021 and LB57, L. jensenii V94G, L. gasseri V105C, and *L. iners* LMG 14328 were cultivated on semisynthetic MRS medium without glucose supplemented with different concentrations of glycogen (i.e., 2%, 1.5%, 1%, 0.75%, 0.5%, 0.25%, 0.1%, and 0.08%). The optical densities (measured at a wavelength of 600 nm) were recorded using a plate reader (BioTek, Winooski, VT, USA). OD was read in intermittent mode, with absorbance readings performed at 3-min intervals for three times after 48 h of growth, where each reading was ahead of 30 s of shaking at medium speed. Cultures were grown in biologically independent triplicates, and the resulting growth data were expressed as the means of these replicates. Glycogen was purchased from Fisher Scientific, ACROS Organics (USA).

### Growth on glycogen as a selection factor for L. crispatus.

For the coculture experiments, MRS medium was used without the presence of glucose yet supplemented with 0.5% of glycogen from beef liver (Fisher Scientific, ACROS Organics, USA). For growth experiments, overnight *Lactobacillus* cultures were diluted to an OD value of 1.0, and overnight Gardnerella vaginalis LMG 7832 culture was diluted to a final concentration that varied between 3 and 4 McFarland units. Each culture was inoculated at 0.1% (vol/vol) into medium. We performed eight different experiments in which L. gasseri V105C, L. jensenii V94G, *L. iners* LMG 14328, and Gardnerella vaginalis LMG 7832 were cultivated together with L. crispatus PRL2021 and LB57 and one experiment where all microorganisms were cocultivated altogether. Batch cultures were incubated under anaerobic conditions for 6 h, 10 h, 24 h, and 30 h at 37°C. After different time points, cultures were centrifuged at 3,000 rpm for 8 min, and the bacterial pellets were harvested. The pellets were subjected to DNA extraction using the GeneElute bacterial genomic DNA kit (Sigma, Germany) following the manufacturer’s instructions. Each sample was subjected to a different cycle of quantitative PCR (qPCR) using species-specific primers: Crisp2_Fw (5′-GGTAATGACGTTAGGAAAGCG-3′) and Crisp33_Rv (5′-GCTGATCATGCGATCTGC-3′) for L. crispatus PRL2021 and LB57, for L. gasseri V105C gassI (5′-GAGTGCGAGAGCACTAAAG-3′) and gassII (5′-CTATTTCAAGTTGAGTTTCTCT-3′), for L. jensenii V94G LjensF (5′-AAGTCGAGCGAGCTTGCCTATAGA-3′) and LjensR (5′-CTTCTTTCATGCGAAAGTAGC-3′) (64), for *L. iners* LMG 14328 LinersF (5′-CTCTGCCTTGAAGATCGGAGTGC-3′) and LinersR (5′-ACAGTTGATAGGCATCATCTG-3′) (65), and for *G. vaginalis* Gard_vaginalis_154-454 FW (5′-CTCTTGGAAACGGGTGGTAA-3′) and Gard_vaginalis_154-454 RV (5′-TTGCTCCCAATCAAAAGCGGT-3′) (66).

qPCR was performed using qPCR green master mix (SensiFAST SYBR No-ROX kit, USA) on a CFX96 system (Bio-Rad, CA, USA) following previously described protocols (66, 67). PCR products were detected with SYBR green fluorescent dye and amplified according to the following protocol: one cycle of 95°C for 2 to 3 min, followed by 40 cycles of 95°C for 15 s and 60 to 65°C for 30 s. The melting curve was 65°C to 95°C with increments of 0.5°C/s. In each run, negative controls (no DNA) were included. A standard curve was built using the CFX96 software (Bio-Rad).

### Glycogen measurement.

Free glycogen in coculture experiments was measured colorimetrically using the glycogen assay kit (BioVision, Milpitas, CA). Ten microliters of supernatants (diluted 1:10) was added to each well in a 96-well microplate with 2 µL of hydrolysis enzyme, and the volume was adjusted to 50 µL with hydrolysis buffer. Samples were incubated according to the manufacturer’s instructions, and absorbance (OD_570 nm_) was measured using a plate reader (BioTek, Winooski, VT, USA).
