# Supplementary material for: Evaluation of Modulatory Activities of Lactobacillus crispatus Strains in the Context of the Vaginal Microbiota
Source: Microbiol Spectr. 2022 Mar 10;10(2):e02733-21. doi: 10.1128/spectrum.02733-21 (PMC9045136; doi:10.1128/spectrum.02733-21)
Supplement: SUPPLEMENTAL FILE 1 — Supplemental material. Download SPECTRUM02733-21_Supp_1_seq6.pdf, PDF file, 0.1 MB [file spectrum02733-21_supp_1_seq6.pdf]

a)

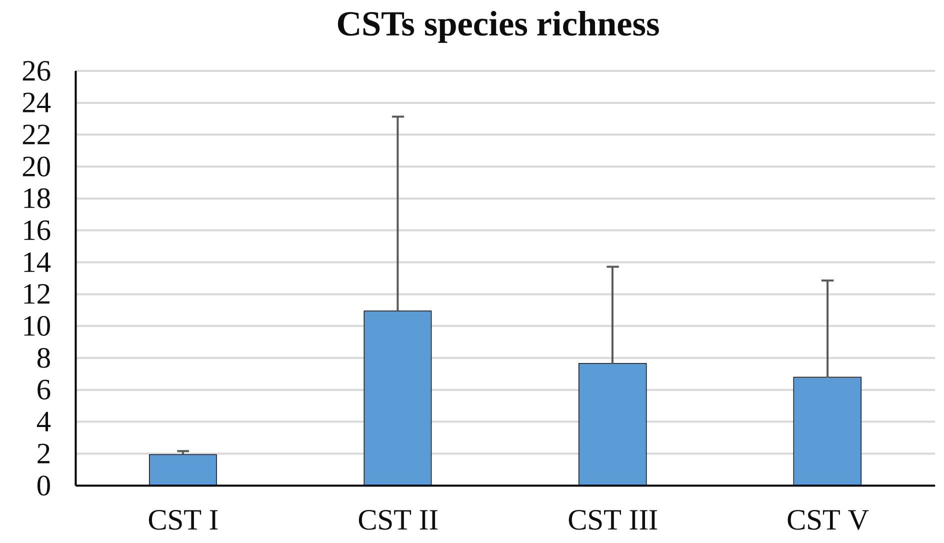
CSTs species richness
26
24
22
20
18
16
14
12
10
8
6
4
2
0
CST I
CST II
CST III
CST V

b)

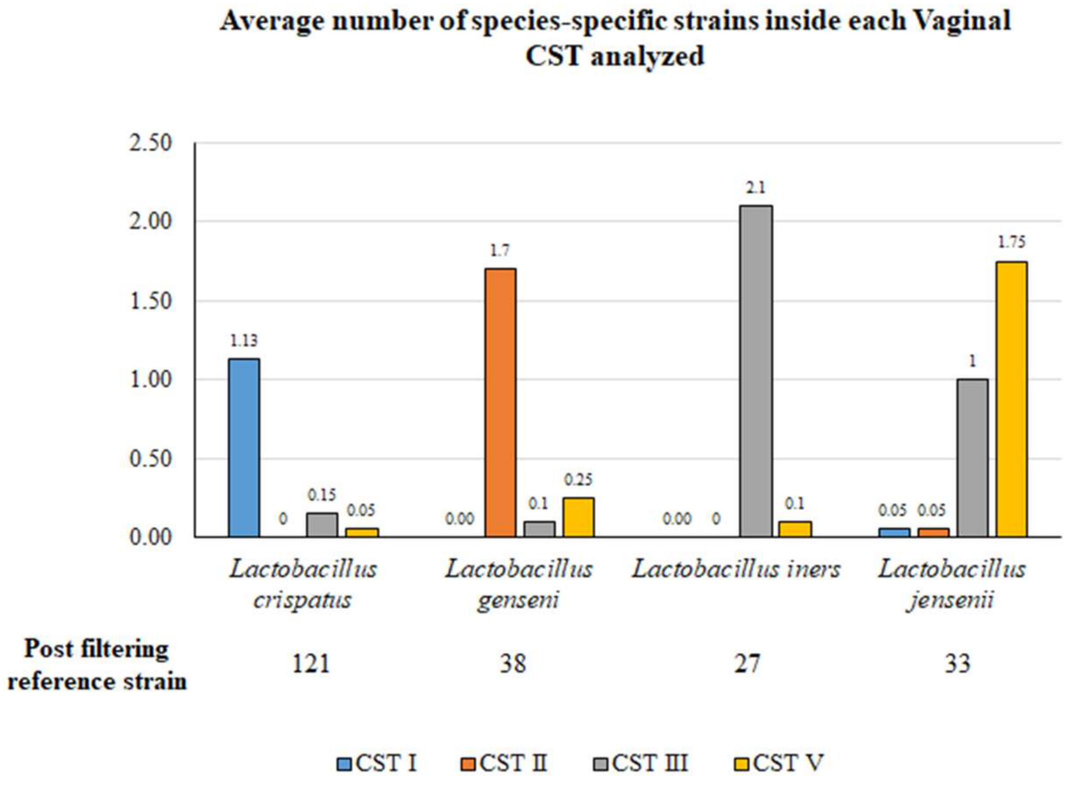
Average number of species-specific strains inside each Vaginal CST analyzed
2.50
2.00
1.50
1.00
0.50
0.00
1.13
0
0.15
0.05
0.00
1.7
0.1
0.25
0.00
0
2.1
0.1
0.05
0.05
1
1.75
Lactobacillus crispatus
Lactobacillus genseni
Lactobacillus iners
Lactobacillus jensenii
Post filtering reference strain
121
38
27
33
CST I
CST II
CST III
CST V

Figure S1

a)

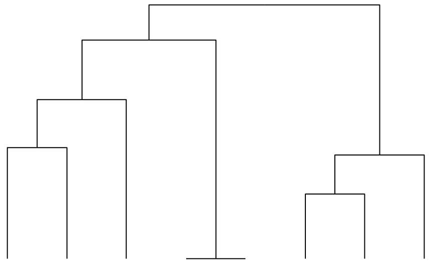

		LCB 1	LCB 6	LCB 2	LCB 7	LCB 8	LCB 3	LCB 5	LCB 4
**Probiotic**	**M247/LB60**	2	1	1	0	0	6	1	0
**Vaginal**	**PRL2021**	1	1	1	0	0	6	0	0
**Vaginal**	**LB56**	1	0	1	0	0	6	0	0
**Vaginal**	**LB57**	2	1	2	0	0	6	1	1
**Vaginal**	**LB58**	2	1	1	0	0	6	1	0
**Vaginal**	**LB59**	2	0	1	0	0	6	1	1
**Vaginal**	**LB61**	2	1	1	0	0	5	1	0
**Vaginal**	**LB62**	2	1	1	0	0	6	1	1
**Vaginal**	**LB63**	2	1	1	0	0	6	1	1
**Chicken**	**LB64**	2	1	2	0	0	0	0	0
**Chicken**	**LB65**	2	1	1	0	0	0	0	0
**Chicken**	**LB66**	2	1	1	0	0	0	0	0
**Chicken**	**LB67**	2	1	2	0	0	0	0	0
**Chicken**	**LB68**	2	1	1	0	0	0	0	0
**Chicken**	**LB69**	2	1	1	0	0	0	0	0
**Chicken**	**LB70**	2	1	1	1	1	6	0	0

b)

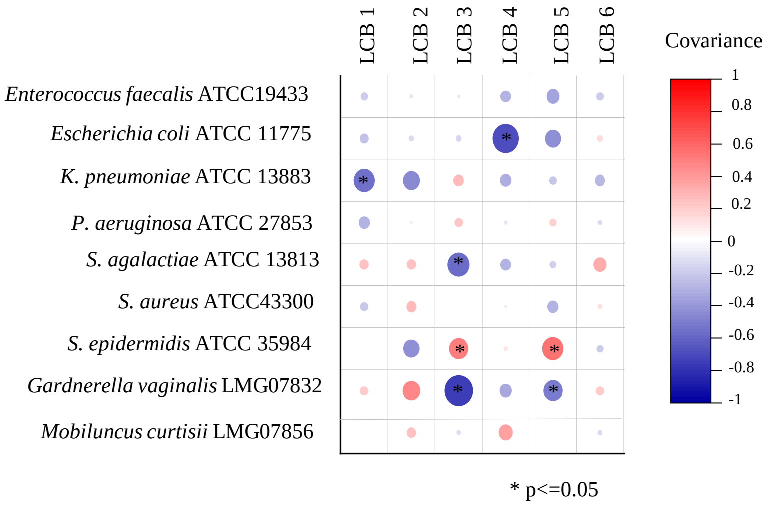

Figure S2

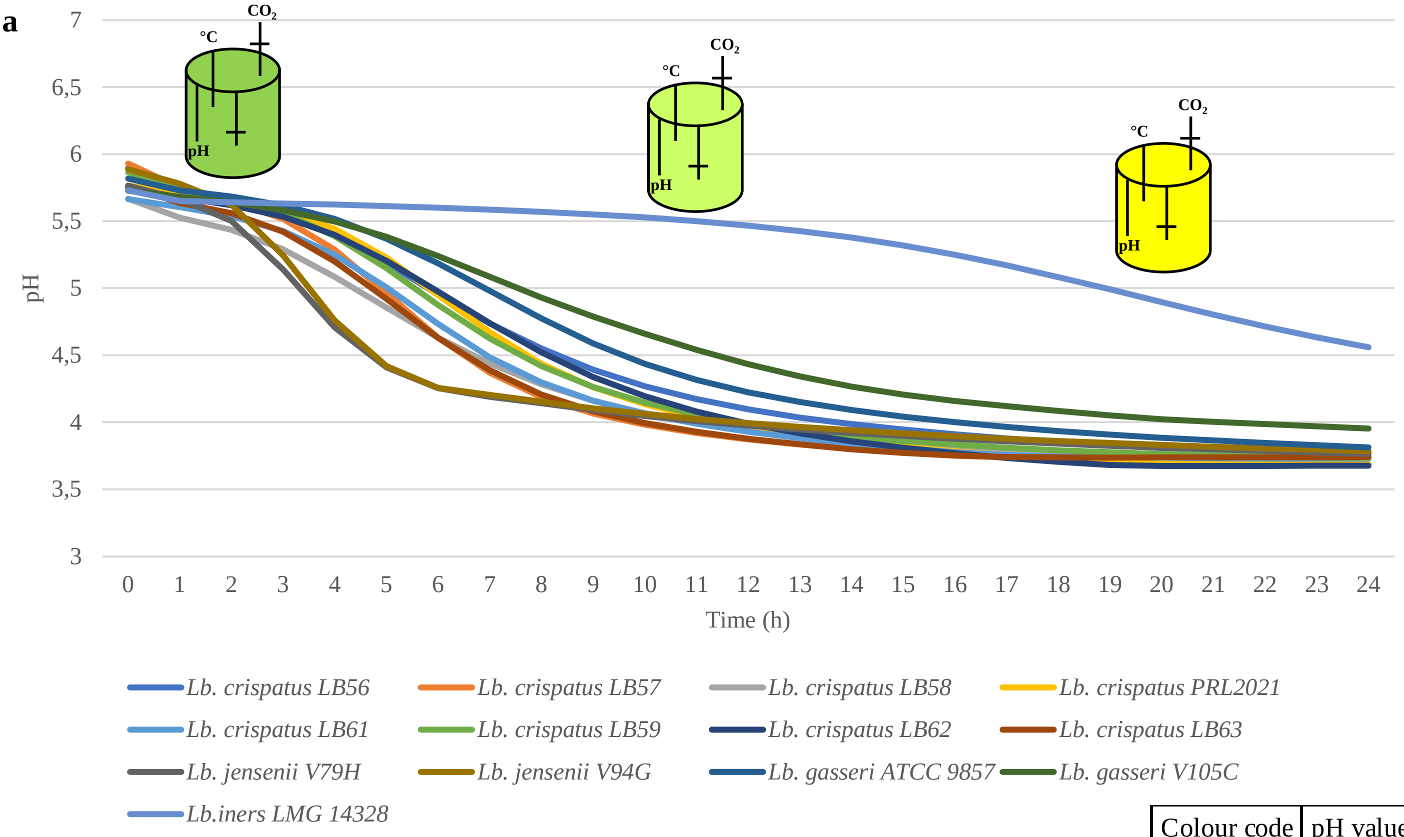

Colour code	pH value
	5.0
	4.0
	3.0

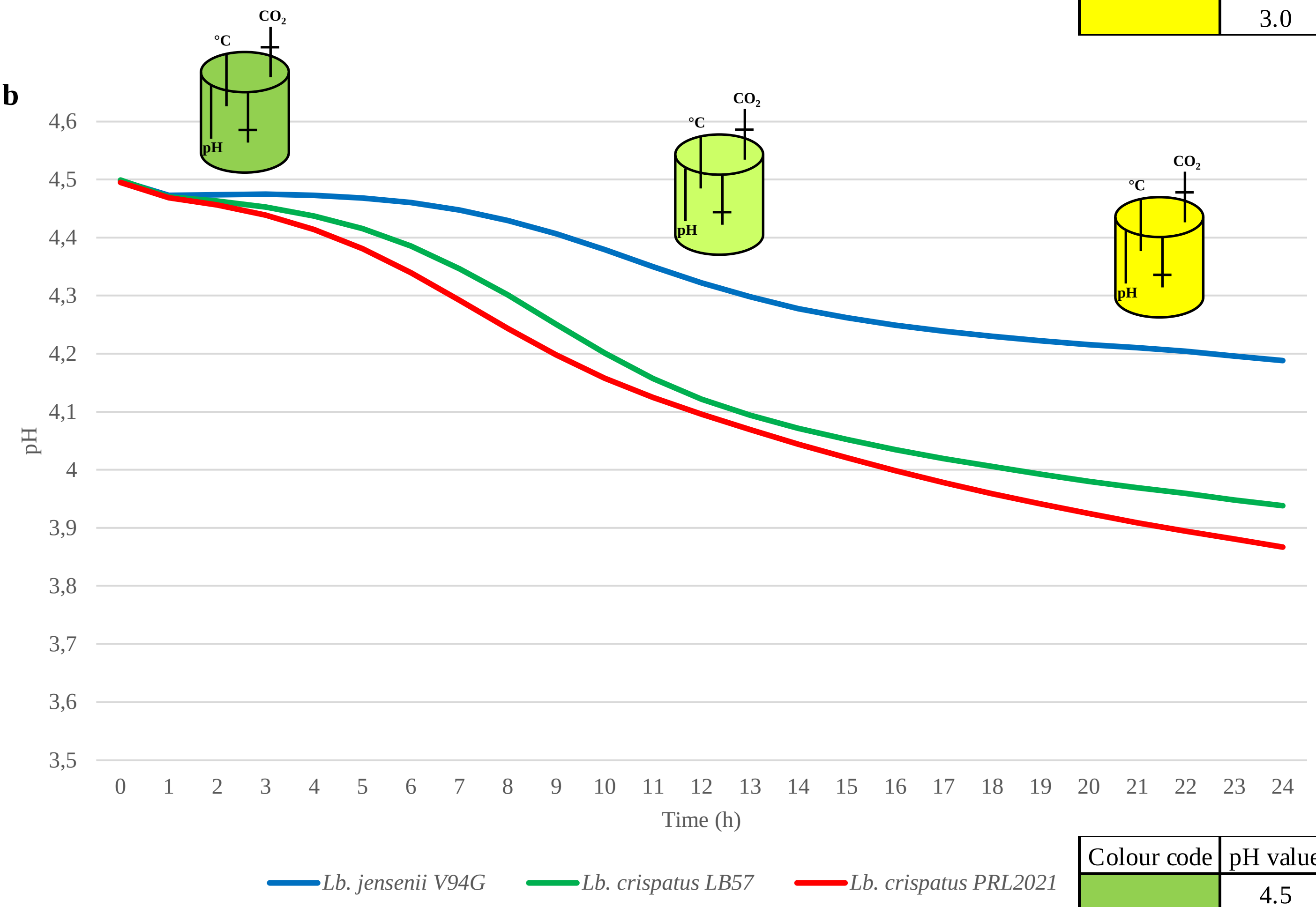

Colour code	pH value
	4.5
	4.3
	4.1

Figure S3

SNPs analysis

DH type	DH1					DH3
Base number	135	655	825	915	942	759
LB62	G	T	T	T	T	A
LB63	G	T	T	T	T	G
LB58	G	T	T	T	T	G
LB59	G	T	T	T	T	G
LB57	A	G	C	C	C	G
LB61	A	G	C	C	C	G
PRL2021	G	G	C	C	C	G
LB56	G	G	C	C	C	G

AA substitution	D	D	D	D	Y	Y	Y	Y
SNPs	T	T	T	T	G	G	G	G
Hours	PRL2021	LB56	LB61	LB57	LB58	LB59	LB63	LB62
0	5.766805	5.89645	5.666625	5.93095	5.66703	5.866285	5.76248	5.75008
1	5.70809	5.73268	5.605935	5.751005	5.525155	5.743165	5.634885	5.67624
2	5.6583	5.6745	5.53725	5.649115	5.43512	5.67178	5.558415	5.618385
3	5.57904	5.57261	5.42669	5.51574	5.2899	5.56089	5.42215	5.533295
4	5.44218	5.40904	5.24976	5.2872	5.085535	5.386605	5.19885	5.396715
5	5.22695	5.18953	5.00504	4.96311	4.854435	5.149875	4.919025	5.204105
6	4.949475	4.95217	4.732485	4.63109	4.63129	4.87417	4.628135	4.974515
Ph Excursion	0.81733	0.94428	0.93414	1.29986	1.03574	0.992115	1.134345	0.775565
7	4.67057	4.73466	4.483935	4.36832	4.43747	4.62313	4.386115	4.739225
8	4.435645	4.54851	4.29602	4.18756	4.28159	4.41868	4.205605	4.520535
9	4.26137	4.39263	4.16063	4.06571	4.161185	4.262245	4.082175	4.337365
10	4.13414	4.26818	4.061655	3.980915	4.06775	4.14523	3.994295	4.19322
11	4.039435	4.17148	3.98761	3.91935	3.995935	4.057	3.92962	4.07945
12	3.96656	4.09486	3.92936	3.8712	3.940075	3.988735	3.87749	3.989475
Ph Excursion	1.800245	1.80159	1.737265	2.05975	1.726955	1.87755	1.88499	1.760605
13	3.90805	4.03474	3.8812	3.832235	3.896345	3.93558	3.835365	3.91764
14	3.860305	3.98509	3.84037	3.79926	3.859285	3.893585	3.797925	3.858235
15	3.819135	3.94451	3.80632	3.77141	3.829005	3.85991	3.771725	3.80925
16	3.784045	3.90874	3.77663	3.75026	3.804535	3.83261	3.75259	3.768415
17	3.75297	3.87842	3.75409	3.738365	3.785305	3.8101	3.74119	3.73382
18	3.725335	3.85147	3.742845	3.741965	3.77208	3.79125	3.737195	3.703765
19	3.702165	3.82818	3.738295	3.746865	3.767125	3.775195	3.736845	3.680825
20	3.68938	3.80656	3.73455	3.751995	3.763135	3.762155	3.736985	3.673875
21	3.687655	3.78752	3.731435	3.756	3.766565	3.75178	3.737775	3.67404
22	3.68785	3.77014	3.728865	3.760015	3.772115	3.74309	3.738765	3.67457
23	3.68693	3.75365	3.728865	3.76109	3.77556	3.73659	3.739235	3.67576
24	3.68556	3.73903	3.72942	3.76165	3.776195	3.73418	3.740185	3.676345
Ph Excursion	2.081245	2.15742	1.93776	2.192585	1.903895	2.132105	2.025635	2.076205

Figure S4

a)

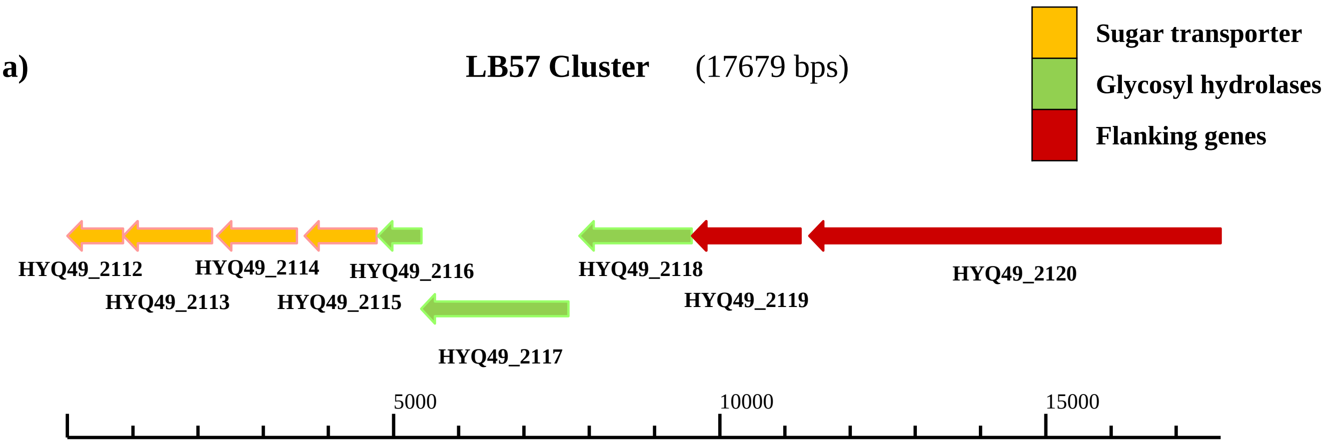

Genes involved in glycogen metabolism

**HYQ49_2120**	–	hypothetical protein
**HYQ49_2119**	–	beta-phosphoglucomutase
**HYQ49_2118**	–	alpha-glycosidase
**HYQ49_2117**	–	maltose phosphorylase
**HYQ49_2116**	–	beta-phosphoglucomutase
**HYQ49_2115**	–	sugar ABC transporter ATP-binding protein
**HYQ49_2114**	–	sugar ABC transporter sugar-binding protein
**HYQ49_2113**	–	sugar ABC transporter permease
**HYQ49_2112**	–	sugar ABC transporter permease

HMMER analysis

Alpha amylase, catalytic domain

Domain E-values

Ind.	Cond.
3.6e-76	3.7e-80

b)

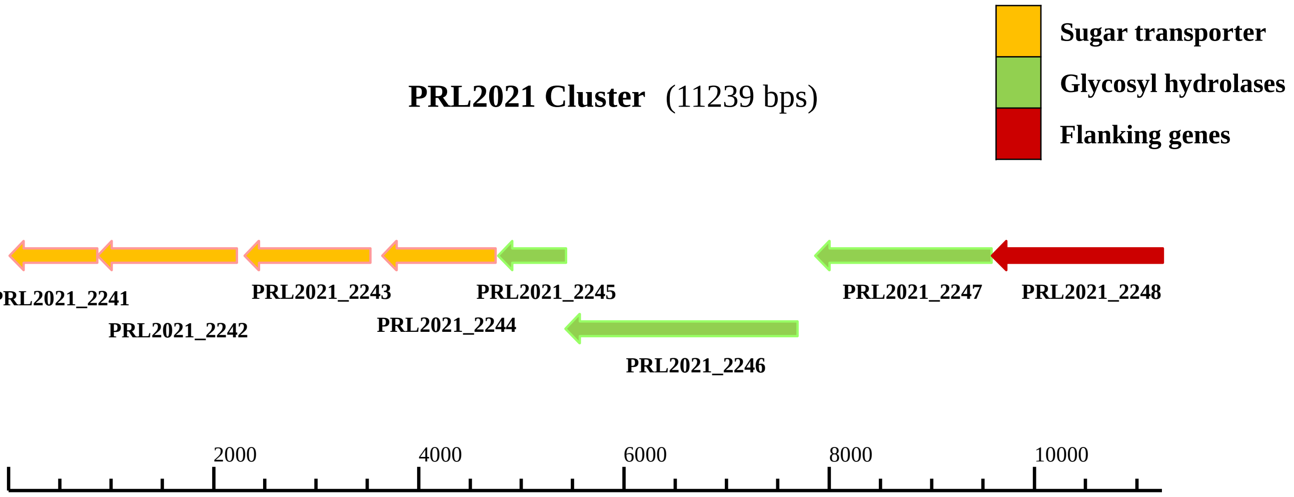

Genes involved in glycogen metabolism

**PRL2021_2248**	–	alpha,alpha-phosphotrehalase
**PRL2021_2247**	–	alpha-glycosidase
**PRL2021_2246**	–	maltose phosphorylase
**PRL2021_2245**	–	beta-phosphoglucomutase
**PRL2021_2244**	–	sugar ABC transporter ATP-binding protein
**PRL2021_2243**	–	maltose ABC superfamily ATP-binding substrate-binding protein
**PRL2021_2242**	–	sugar ABC transporter permease
**PRL2021_2241**	–	sugar ABC transporter permease

FigureS5

Supplementary figure legends

Figure S1. Microbial biodiversity in the vaginal microbiota.

Panel a reports the species richness analysis obtained through METAnnotatorX2 profiling of the 80 vaginal samples. Panel b shows the strain tracking result for the 4 *Lactobacillus* species analyzed in 80 vaginal samples, divided by CST I, II, III and V, through the use of strainGST software.

Figure S2. Analysis of bacteriocin gene distribution and correlation to inhibition halo.

Panel a reports an HCl analysis relative to the distribution of the six bacteriocin clusters (LCB) between the analyzed strains of *Lb. crispatus*, while panel b) shows correlation of the presence of the six different LCB and the inhibition halo of *Lb. crispatus* grown with the nine species used as test.

Figure S3. Evaluation of lactic acid production by vaginal lactobacilli.

Panel a displays the pH lowering through fermentation process in MRS. The variation of pH as a function of time is reported. Panel b) shows the evaluation of lactic acid production through fermentation process in Simulated Vaginal Fluid (SVF). The variation of pH as a function of time is reported.

Figure S4. SNP analysis in DH genes.

The figure reports all five SNPs identified in the DH1 gene and the single SNP identified in the DH3 gene, with the relative NT substitution. Additionally, for the SNP at position 655 in the DH1 gene, is reported also the relative AA subsitution, correlated to pH profile of each *Lb. crispatus* genome isolated from human vagina.

Figure S5. Cluster of genes for glycogen metabolism in *Lactobacillus crispatus*.

The figure shows the cluster of genes involved in glycogen metabolism conserved within the *Lactobacillus crispatus* strains used in the growth assays. Panels a) and b) show the genes present within the conserved cluster for glycogen metabolism both in LB57 and PRL2021 strains.
